# Successful Treatment of Plaque Psoriasis with Allogeneic Gingival Mesenchymal Stem Cells: A Case Study

**DOI:** 10.1155/2020/4617520

**Published:** 2020-03-28

**Authors:** Sebo Gene Wang, Nicholas C. Hsu, Sebo Michelle Wang, Fu Nan Wang

**Affiliations:** ^1^Top IVF USA, Hacienda Heights, CA, USA; ^2^Loma Linda University School of Medicine, Loma Linda, CA, USA; ^3^Stem Cell Life Science Corp., New Taipei City, Taiwan

## Abstract

Plaque psoriasis is the most common type of psoriasis that manifests as red scaly patches with white scales affecting body areas including scalp, elbows, knees, trunk, and buttocks. Although many treatment options are available including novel biologics, no cure is available. Mesenchymal stem cells (MSCs) have been safely used to treat a variety of human diseases. Allogeneic MSCs possess unique characteristics including hypoimmunogenicity, immunomodulatory, and anti-inflammatory properties, and they are currently being explored for potential therapeutic use for many systemic inflammatory diseases. The human gingival tissue is an easily accessible and obtainable source for the isolation of MSCs. MSCs from adult human gingiva are of fetal-like phenotype, multipotent, and easy to isolate and expand *in vitro*. Herein, we report a case of a 19-year-old man with a 5-year history of severe plaque psoriasis refractory to multiple topical and systemic therapies who was treated with allogeneic human gingival MSCs. Complete regression was achieved after 5 infusions with no adverse reaction occurred. The patient has been followed for three years and has remained disease free.

## 1. Introduction

Plaque psoriasis, the most common variant of psoriasis, makes up approximately 85 to 90 percent of all psoriasis cases [1]. This form of psoriasis typically presents with raised red scaly patches covered with white scales. These lesions/plaques most commonly occur on scalp, extensor surfaces of the elbows and knees, trunk, and buttocks, though it can appear on any location [2]. Psoriasis is associated with several other serious health conditions, such as diabetes, cardiovascular disease, lymphoma, and depression [2–4]. The etiology of psoriasis included genetic and environmental factors, yet it is complex and not well understood. Population and genetic studies have revealed a strong hereditary component, and the mode of inheritance is multifactorial [5–7]. Many environmental, physiologic, and psychological factors including infections, skin trauma, alcohol, tobacco, obesity, medications, and stress are known to trigger psoriasis and/or exacerbate existing condition [8]. Treatment options are diverse and range from topical medications including salicylic acid, coal tar, anthralin, corticosteroids, vitamin D3 analogs, calcineurin inhibitors, and tazarotene for mild disease, UV light therapy for moderate disease, and systemic agents such as methotrexate, oral retinoids, cyclosporine, and biologics for more severe disease [9]. Although these conventional therapies are effective for symptom control, there is no permanent cure for psoriasis [2, 9].

Present in virtually all tissues, mesenchymal stem cells (MSCs) are multipotent adult stem cells that possess the ability of self-renewal and multilineage differentiation [10–12]. MSCs can be readily isolated and expanded in vitro [13, 14] and, thus, hold great therapeutic potential in both autologous and allogeneic settings. Although autologous transplantation is a safe therapeutic option in the context of immunogenicity, the quantity, quality, and proliferation capability of MSCs were often reduced due to age and existing diseases of the patients [15–20]. MSCs are inherently hypoimmunogenic due to low-level major histocompatibility complex (MHC) class I expression and the lack of expression of MHC class II and co-stimulatory molecules [21, 22]. These unique immunosuppressive properties render them safe for allogeneic use [23–25]. The gingiva, composed of epithelium and connective tissues, is a unique biological barrier of the human periodontium. One of the gingiva's distinct characteristics is its fast wound healing ability following injury with little or no scarring [26]. Readily accessible and only minimally invasive procedure is needed for sampling, and this tissue represents an ideal source of multipotent postnatal stem cells for cell-based therapy in regenerative medicine. Here, we report a case of severe plaque psoriasis successfully treated with allogeneic gingiva-derived (G-) MSCs.

## 2. Case Report

**Figure 1:**
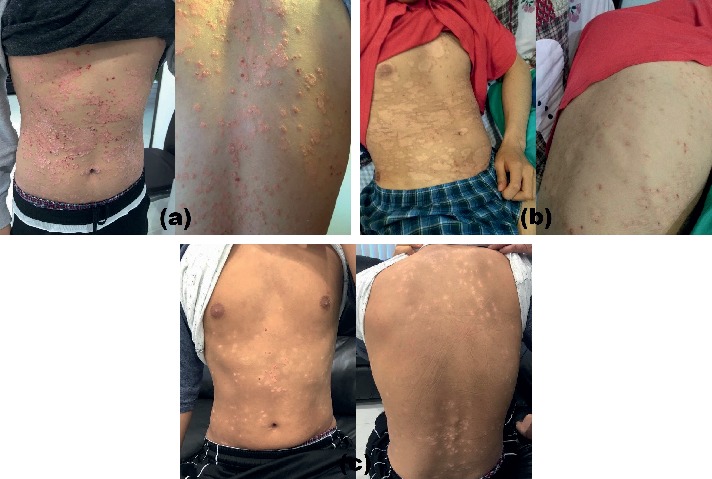
The effects of G-MSC infusions on a 19-year-old man with a 5-year history of severe plaque psoriasis. (a) Baseline/preinfusion images showing demarcated, round, erythematous plaques on upper chest and abdomen. (b) Gradual clearing of plaques after two successive weekly administrations of MSCs. (c) Lesions fully cleared after three more MSC infusions.

A 19-year-old male college student presented with a 5-year history of severe plaque psoriasis. Physical examination revealed demarcated, round, erythematous plaques with overlying thick white scales distributed throughout his body (Figure 1(a)). The patient experienced itching often and described the intensity as unbearable. This disease had not only taken a toll on the patient physically, but he also suffered from depression and had suicidal thoughts. The patient had received several systemic treatments including methotrexate, acitretin, cyclosporine, and etanercept but with limited response. Because of the severity of the disease and lack of treatment response to prior systemic therapies, allogeneic MSC-based therapy was offered as an option based on earlier reports of success. The patient and the family were well informed regarding the experimental nature of MSC-based therapy. Written informed consent for treatment and data publication was obtained from the patient. The patient had not received phototherapy or other treatments during and after the MSC-based therapy.

Isolation and expansion of MSCs were performed according to the method previously described by Mitrano et al. [27]. Briefly, a biopsy of gingival connective tissue was obtained from a healthy adult donor. The tissue was minced for explant culture. The resulting cells were harvested and expanded in vitro for 3 to 4 weeks. Afterwards, the cells were aliquoted and cryopreserved. On days of treatment, the MSCs were thawed and made ready for administration. MSCs were suspended in 5  mL of normal saline prior to bolus injection. The patient received two successive weekly administrations of MSCs (3 × 10^6^/Kg/infusion). Gradual clearing of plaques was observed, and no adverse effect was reported by patient (Figure 1(b)). Five weeks later, we gave the patient three more weekly MSC infusions. A week after the last injection, his psoriatic lesions fully cleared (Figure 1(c)). The patient has been free of psoriasis for 3 years now.

## 3. Discussion

With roughly 2% of population affected, psoriasis is a major public health burden worldwide [28]. Patients with psoriasis frequently experience comorbidities and are at higher risk for depression and suicide [2–4]. Although an array of treatment options exists, an effective and durable therapy has yet been developed. Psoriasis is an immune-mediated chronic systemic inflammatory disease. Because MSCs exert potent anti-inflammatory and immunomodulatory effects and possess important immunoevasive properties, there has been growing interest in the use of these cells as therapeutics for psoriasis [29–31]. G-MSCs form adult gingival mucosa which are of fetal-like phenotype and multipotent and possess strong immunomodulatory properties [32]. G-MSCs have notably several advantages as a candidate cell source over other MSCs. These cells are homogeneous and proliferate faster than other mesenchymal stem cells. G-MSCs also display stable morphology, maintain normal karyotype and telomerase activity at high passages [33]. Moreover, G-MSCs cells are abundant and easily accessible and can been effectively isolated and expanded *in vitro* for clinical use. To the best of our knowledge, this is the first study to report the safety and the effects of repeated administration of G-MSCs in a patient with psoriasis. The patient has been monitored for three years and has remained relapse free. This novel treatment vastly improved the quality of life and the psychological well-being of the patient. The findings of this study were in agreement with previous reports with regards to safety and feasibility of MSC infusions in patients with severe psoriasis [30, 31]. Therapeutic application of MSCs may be potentially superior to conventional clinical treatment modalities for severe psoriasis. Further studies that involved a larger sample size are needed to optimize key components of MSC therapies such as infusion frequency, method, and dosage.
